# mitoSomatic: a tool for accurate identification of mitochondrial DNA somatic mutations without paired controls

**DOI:** 10.1002/1878-0261.13335

**Published:** 2022-12-15

**Authors:** Wenjie Guo, Yang Liu, Liping Su, Shanshan Guo, Fanfan Xie, Xiaoying Ji, Kaixiang Zhou, Xu Guo, Xiwen Gu, Jinliang Xing

**Affiliations:** ^1^ State Key Laboratory of Cancer Biology and Department of Physiology and Pathophysiology Fourth Military Medical University Xi'an China; ^2^ State Key Laboratory of Cancer Biology, Department of Pathology, Xijing Hospital and School of Basic Medicine Fourth Military Medical University Xi'an China

**Keywords:** machine learning, mitochondrial DNA, next‐generation sequencing, somatic mutations

## Abstract

Mitochondrial DNA (mtDNA) somatic mutations play important roles in the initiation and progression of cancer. Although next‐generation sequencing (NGS) of paired tumor and control samples has become a common practice to identify tumor‐specific mtDNA mutations, the unique nature of mtDNA and NGS‐associated sequencing bias could cause false‐positive/‐negative somatic mutation calling. Additionally, there are clinical scenarios where matched control tissues are unavailable for comparison. Therefore, a novel approach for accurately identifying somatic mtDNA variants is greatly needed, particularly in the absence of matched controls. In this study, the ground truth mtDNA variants orthogonally validated by triple‐paired tumor, adjacent nontumor, and blood samples were used to develop mitoSomatic, a random forest‐based machine learning tool. We demonstrated that mitoSomatic achieved area under the curve (AUC) values over 0.99 for identifying somatic mtDNA variants without paired control in three tumor types. In addition, mitoSomatic was also applicable in nontumor tissues such as adjacent nontumor and blood samples, suggesting the flexibility of mitoSomatic's classification capability. Furthermore, analysis of triple‐paired samples identified a small group of variants with uncertain somatic/germline origin, whereas application of mitoSomatic significantly facilitated the prediction of their possible source. Finally, a control‐free evaluation of the public pan‐cancer NGS dataset with mitoSomatic revealed a substantial number of variants that were probably misclassified by conventional tumor‐control comparison, further emphasizing the usefulness of mitoSomatic in application. Taken together, our study demonstrates that mitoSomatic is valuable for accurately identifying somatic mtDNA variants in mtDNA NGS data without paired controls, applicable for both tumor and nontumor tissues.

## Graphical abstract

Mitochondrial DNA (mtDNA) somatic mutations play important roles in cancer. Due to mtDNA heterogeneity, conventional NGS sequencing strategy of paired tumor and control samples is flawed and inapplicable for scenarios without matched normal tissue. We present mitoSomatic, a novel random forest‐based machine learning tool, to accurately identify mtDNA somatic mutations in mtDNA NGS data without paired controls, applicable for both tumor and nontumor tissues.

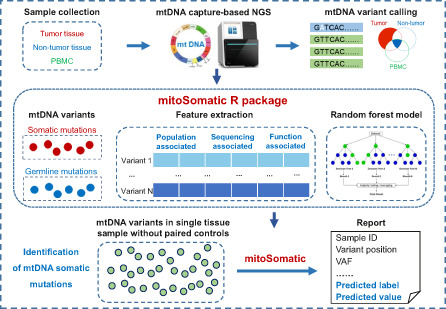

**Abbreviations**
AUC: area under the curve
CRC: colorectal cancer
dbSNP: the single nucleotide polymorphism database
HCC: hepatocellular carcinoma
mtDB: human mitochondrial genome database
mtDNA: mitochondrial DNA
nDNA: nuclear DNA
NGS: next‐generation sequencing
NUMTs: nuclear mitochondrial DNA segments
PBMC: peripheral blood mononuclear cell
RCC: renal cell carcinoma
RF: random forest
SVM: support vector machine
VAF: variant allele frequency
WES: whole‐exome sequencing
WGS: whole‐genome sequencing

## 1 Introduction

Mitochondria contain their unique genome, the mitochondrial DNA (mtDNA), which in humans is a genetically compact, double‐stranded, circular molecular of 16.5 kb, encoding 2 rRNAs, 22 tRNAs, and 13 proteins essential for oxidative phosphorylation [1]. Unlike diploid nuclear DNA (nDNA), mtDNA had an exclusive maternal origin and contained hundreds of copies in one cell [2]. Owing to the lack of protective histones and limited DNA repair activities, mtDNA has a 10‐fold higher mutation rate than nDNA, which contributes to a high abundance of mtDNA polymorphisms (germline) in the population and a high incidence of somatic mtDNA mutagenesis [3]. In principle, germline mtDNA variants are maternally inherited and shared in most or all tissues, whereas somatic mtDNA variants arise as *de novo* mutations in specific tissues or organs [4]. Accumulating evidence from large cohort pan‐cancer studies indicates that somatic mtDNA variants are prevalent in tumor cells [5, 6] and play essential roles in cancer biology, including tumorigenesis [7, 8], metastasis [9], and prognosis [10]. Thus, accurate identification of somatic mtDNA variants is of great importance in cancer studies.

The advent of next‐generation sequencing (NGS) has dramatically improved the sensitivity of mtDNA variant calling, with variant allele frequency (VAF) down to 1% reliably detected [6, 11, 12]. In previous studies, either matched adjacent nontumor or peripheral blood mononuclear cell (PBMC) samples were commonly used as normal controls to identify somatic mutations in tumor tissues, in which somatic mutations were defined as tumor‐specific variants, while variants shared by both tissues were treated as germline [5, 13]. However, recent profiling of mtDNA variants across different normal tissues has revealed the presence of mtDNA variants with uncertain germline or somatic origin. Because such mtDNA variants can be observed in only two tissue types but are absent in other tissues [14]. These findings suggest that some mtDNA ‘germline variants’ determined based on paired normal and tumor samples may be false positive or misleading. Furthermore, there are commonly encountered scenarios where matched control tissues are not available, thus hindering the identification of somatic and germline mtDNA variants in tumor tissue. For instance, many fresh frozen tumor samples have no matched control tissues available in retrospective clinical studies. Therefore, a novel method for the accurate identification of somatic mtDNA variants in tumor tissue is greatly needed, particularly in situations where matched control is unavailable.

Several bioinformatic tools have been developed to identify somatic mutations of nuclear DNA based on matched tumor and nontumor samples, including MutationSeq [15], SNooPer [16], SomaticSeq [17], and DeepSSV [18]. Still, these tools mainly focused on the exclusion of sequencing artifacts. Recently, a few studies have attempted to identify somatic and germline variants in nuclear DNA without matched controls, such as ISOWN [19] and SomVarIUS [20]. Nevertheless, these methods are unsuitable for identifying somatic mtDNA variants with or without matched controls due to significant differences between mtDNA and nuclear DNA variants.

In this study, based on experimentally validated somatic mutations, we developed a random forest‐based machine learning tool, mitoSomatic, to accurately identify somatic mtDNA variants without paired controls. This tool may facilitate a better profiling of somatic mtDNA variants in both malignant and nonmalignant tissues, consequently promoting a better understanding of somatic mtDNA variants in the initiation and progression of cancer.

## 2 Materials and methods

### 2.1 Patient specimen

A total of 157 hepatocellular carcinoma (HCC), 24 colorectal cancer (CRC), and 18 renal cell carcinoma (RCC) patients who had undergone surgery and had no treatment before collection of tissue samples were enrolled between August 2015 and June 2018 from Xijing Hospital, Fourth Military Medical University (FMMU, Xi'an, China). Diagnosis of HCC, CRC, and RCC was confirmed by histological pathology. This study was approved by the Ethics Committee of FMMU (KY20183331‐1), and written informed consent was obtained from all patients complying with the Declaration of Helsinki. Triple‐paired tumor, nontumor, and PBMC tissue samples were collected from each patient, where nontumor tissues were obtained at least 2 cm away from the tumor margin. Notably, the adjacent nontumor tissues in the HCC cohort were predominantly inflammatory or cirrhosis liver tissues. Two independent pathologists carefully reviewed hematoxylin and eosin (HE) staining slides to ensure that the cancer cell content was > 90% in tumor tissues and there was no contamination of tumor cells in nontumor tissues.

### 2.2 DNA extraction, library construction, and capture‐based mtDNA sequencing

The genomic DNA was extracted from the isolated peripheral blood mononuclear cells (PBMCs) and fresh tissue samples using ENZA DNA Kit (Omega, Norcross, GA, USA). DNA quality and quantity were assessed using a 2100 bioanalyzer (Agilent Technologies, Santa Clara, CA, USA) and qubit (Invitrogen, Carlsbad, CA, USA), respectively. The sequencing library for the Illumina platform was constructed as previously described [21]. In brief, 1 μg genomic DNA from PBMCs and fresh tissue samples was sonicated randomly by a focused ultrasonicator (Scientz98, Ningbo, China) to obtain fragments between 300 and 500 bp in length. DNA fragments were end‐repaired, ligated with sequencing adapters, and PCR amplified for nine cycles. Then, mtDNA fragments were captured by hybridization using homemade biotinylated mtDNA probes as previously described [10, 22]. Finally, the captured mtDNA libraries were amplified and sequenced on a Illumina Hiseq 2500 platform using paired‐end runs (2 × 125 cycles).

### 2.3 mtDNA variant calling pipeline

An innovative pipeline previously established in our laboratory was used for accurate mtDNA variant calling [22]. Briefly, raw mtDNA sequencing reads were aligned to the revised Cambridge Reference Sequence (rCRS) and hg19 with BWA [23] to eliminate contamination from nuclear DNA of mitochondrial origin (NUMT). After sorting and removing duplicated reads with the Picard tool, local realignment was performed with IndelRealigner in GATK software to reduce the false‐positive rate near indel positions [24]. Finally, we selected high‐quality reads by SAMtools for further analysis [23]. A series of subsequent filter conditions were also used to call mtDNA variants, including (i) Variant allele frequency (VAF) ≥ 1%; (ii) at least 100× depth on each variant site; (iii) base quality ≥ 30; (iv) at least three reads on each strand supporting the variant site; (v) removing heterogeneity sites in rCRS repeat regions (66–71, 303–311, 514–523, 12 418–12 425, 16 184–16 193); (vi) removing C > A/G > T mutations with strong sequence context bias (at CpCpN > CpApN; most frequently at CpCpG > CpApG) and low VAF (1%–2%) due to artificial guanine oxidation [25]. Moreover, sample cross‐contamination was also evaluated, and contamination‐derived variants were filtered by the machine learning approach we developed [26].

### 2.4 Workflow of mitoSomatic

**Fig. 1:**
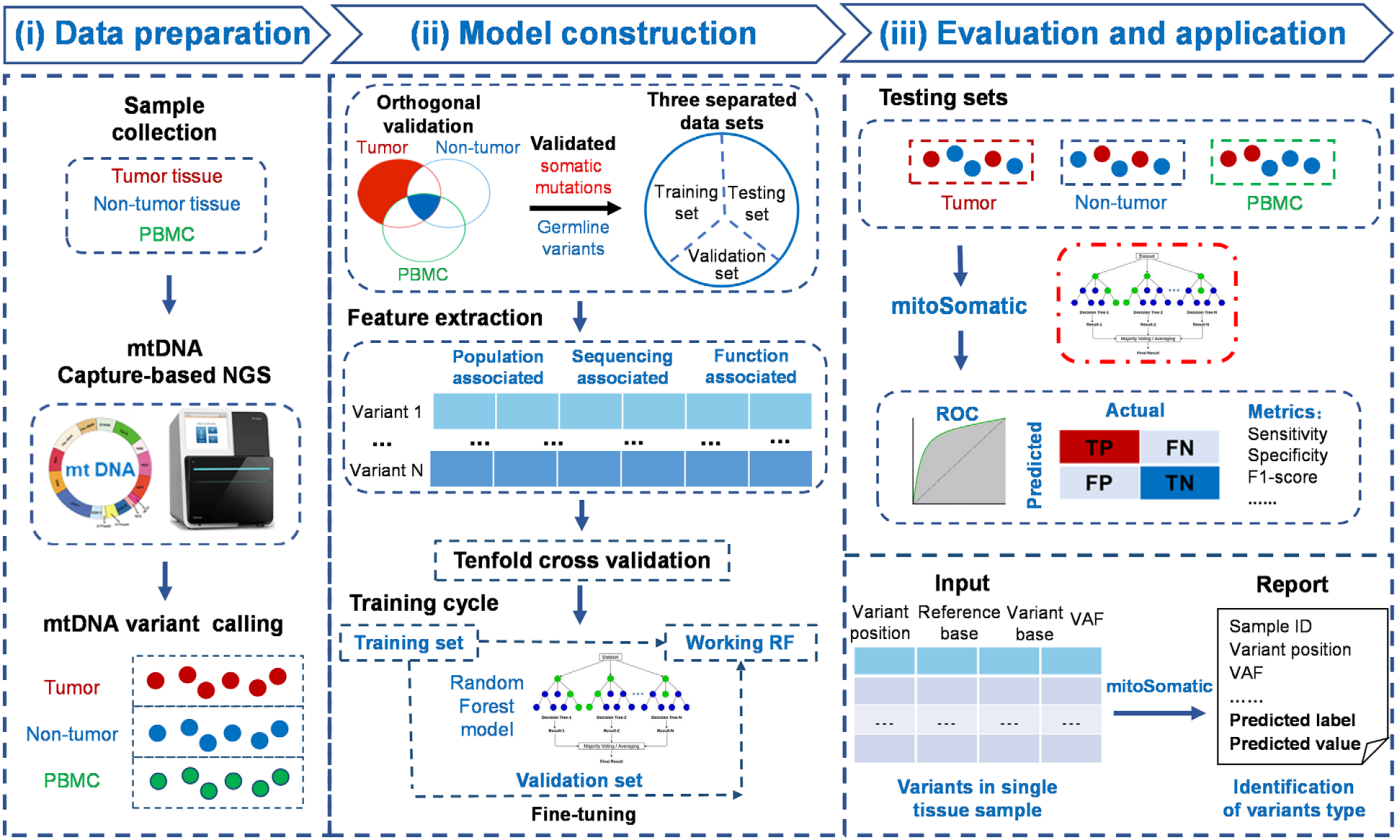
Workflow of mitoSomatic. mitoSomatic framework consists of data preparation, model construction, and model evaluation and application. (i) Capture‐based NGS of mtDNA was performed for tumor, adjacent nontumor, and PBMC samples to identify mtDNA variants. (ii) Ground truth somatic and germline mtDNA variants were generated by orthogonal validation of triple‐paired samples, which were randomly split into three exclusive datasets. The random forest model was selected as the optimal classification algorithm by tenfold cross‐validation. (iii) mitoSomatic performance was evaluated by six testing sets derived from different tumor and tissue types. mtDNA variants were input into the mitoSomatic package to report the variants type. FN, false negative; FP, false positive; NGS, next‐generation sequencing; PBMC, peripheral blood mononuclear cell; RF, random forest; TN, true negative; TP, true positive; VAF, variant allele frequency.

mitoSomatic is a machine learning tool for accurately identifying mtDNA somatic mutations in a specific tissue sample without control (Fig. 1). First, ground truth somatic and germline mtDNA variants, generated by orthogonal validation of triple‐paired samples, were split into three exclusive datasets: training, validation, and testing sets. As the optimal classification algorithm, the random forest model was then developed with the training set, with parameters fine‐tuned by evaluating the performance with the validation set. Finally, mitoSomatic performance was evaluated by six testing sets. A tab‐formatted mtDNA variant table was used as the input file for the mitoSomatic package to report the variant type.

### 2.5 Somatic and germline mtDNA variant data

Triple‐paired tumor, adjacent nontumor, and PBMC tissue samples from 157 hepatocellular carcinoma (HCC), 24 colorectal cancer (CRC), and 18 renal cell carcinoma (RCC) patients were used to generate the somatic and germline mtDNA variant data in our laboratory. DNA extraction, library construction, capture‐based mtDNA NGS sequencing, and subsequent mtDNA variant calling were performed according to previously established pipelines [10, 21, 22, 27]. The sequencing data summary is shown in Table S1. Variants detected are shown in Fig. S1 and Table S2 (T, P, and B denote tumor, adjacent nontumor, and PBMC samples, respectively). To avoid false‐positive variants due to sequencing error or contamination of nuclear mitochondrial DNA segments (NUMTs), we only reported variants with VAF ≥ 1%. In this study, ground truth mtDNA somatic mutations were defined as variants present only in one sample, while ground truth germline variants were defined as variants present in all three samples. Those variants present in only two of three paired samples were defined as variants of uncertain origin. Variants of uncertain origin in 157 HCC patients were shown in Table S3.

Ground truth somatic and germline mtDNA variants in the paired tumor, adjacent nontumor, and PBMC samples from 157 HCC, 24 CRC, and 18 RCC patients were used to generate training, validation, and testing sets (Table S4). Public mtDNA somatic and germline variant data from whole‐genome sequencing (WGS) or whole‐exome sequencing (WES) data of pan‐cancer tumor samples were directly downloaded from one published study, *eLife* 2014 [5], and used for mitoSomatic evaluation.

### 2.6 Feature selection

For each variant, nine features for differentiating somatic and germline mtDNA variants were extracted, including four population‐associated, three sequencing‐associated, and two function‐associated features. The complete list of features and descriptions is presented in Table S5. weka‐InfoGain (version 3.8) feature selection tool was used to ensure all the selected features were informative and not redundant.

### 2.7 Tenfold cross‐validation

To determine the optimal learning algorithm, six representative supervised learning algorithms provided by weka (version 3.8.4) [28], including J48DT, JRip, Naive Bayes, random forest, support vector machine (SVM), and logistic regression, were evaluated by tenfold cross‐validation with default parameters, which was carried out based on 263 ground truth somatic and 5251 germline variants in 157 HCC tumor samples. The optimal classification algorithm was determined by six main evaluation metrics, including area under the curve (AUC), accuracy, sensitivity, specificity, precision, and F1‐score.

### 2.8 Random forest model

The random forest algorithm, outperforming others in tenfold cross‐validation, was further optimized to establish mitoSomaitc. The optimization of the training set for mitoSomatic was performed based on training sets 1–2, and validation sets 1–2. mitoSomatic was trained using variants selected from training set 1 and validated by validation set 1 to evaluate the effect of the sample size and somatic and germline mtDNA variants ratio in the training set. mitoSomatic was trained using one cancer type (training set 1 or 2) and then tested on the same or another cancer type (validation sets 1 and 2) to evaluate the effect of the cancer type in the training set. Model training was performed using the r package ‘RandomForest’ (version 4.6–14). Finally, we developed a primary model with training set 1. To achieve optimal performance, multiple learning cycles were evaluated using the validation set 1 to fine‐tune the training process and parameter settings, including mtry (the number of variables randomly sampled as candidates at each split) and ntree (the number of trees). The importance of each feature was calculated in the optimal model based on training set 1.

### 2.9 Evaluation and application of mitoSomatic

Testing sets 1 and 2 were used to evaluate the performance of mitoSomatic in tumor tissues. Testing sets 3–6 were used to assess the capability of mitoSomatic to identify somatic mtDNA variants in adjacent nontumor and PBMC samples. In addition, a public dataset from pan‐cancer tumor samples was also used to evaluate mitoSomatic performance.

### 2.10 Code implementation and output of mitoSomatic

mitoSomatic was edited in the r programming language, in which a few external databases are required, including Mitomap [29], dbSNP v155 [30], annovar [31], mtDB [32], and Mutation assessor [33]. The output of mitoSomatic is a tab‐delimited text report, including the sample name, variant position, and nine primary features for each mtDNA variant, with additional columns describing the predicted variant type (somatic or germline) and the probability (ranging from 0 to 1) of being somatic mutations. Furthermore, the user can manually check this report to evaluate whether the predicted somatic mutations are reliable.

### 2.11 Statistical analysis

graphpad prism 8.0 (GraphPad, La Jolla, CA, USA) was used for statistical analysis. The Chi‐square test was used for comparing groups with categorical variables. The two‐sample Kolmogorov–Smirnov test was used to test whether two samples come from the same distribution. All *P* values were two‐tailed and reported using a significance level of 0.05.

## 3 Results

### 3.1 VAF distribution and the occurrence in human population for mtDNA somatic and germline variants in tumor samples

**Fig. 2:**
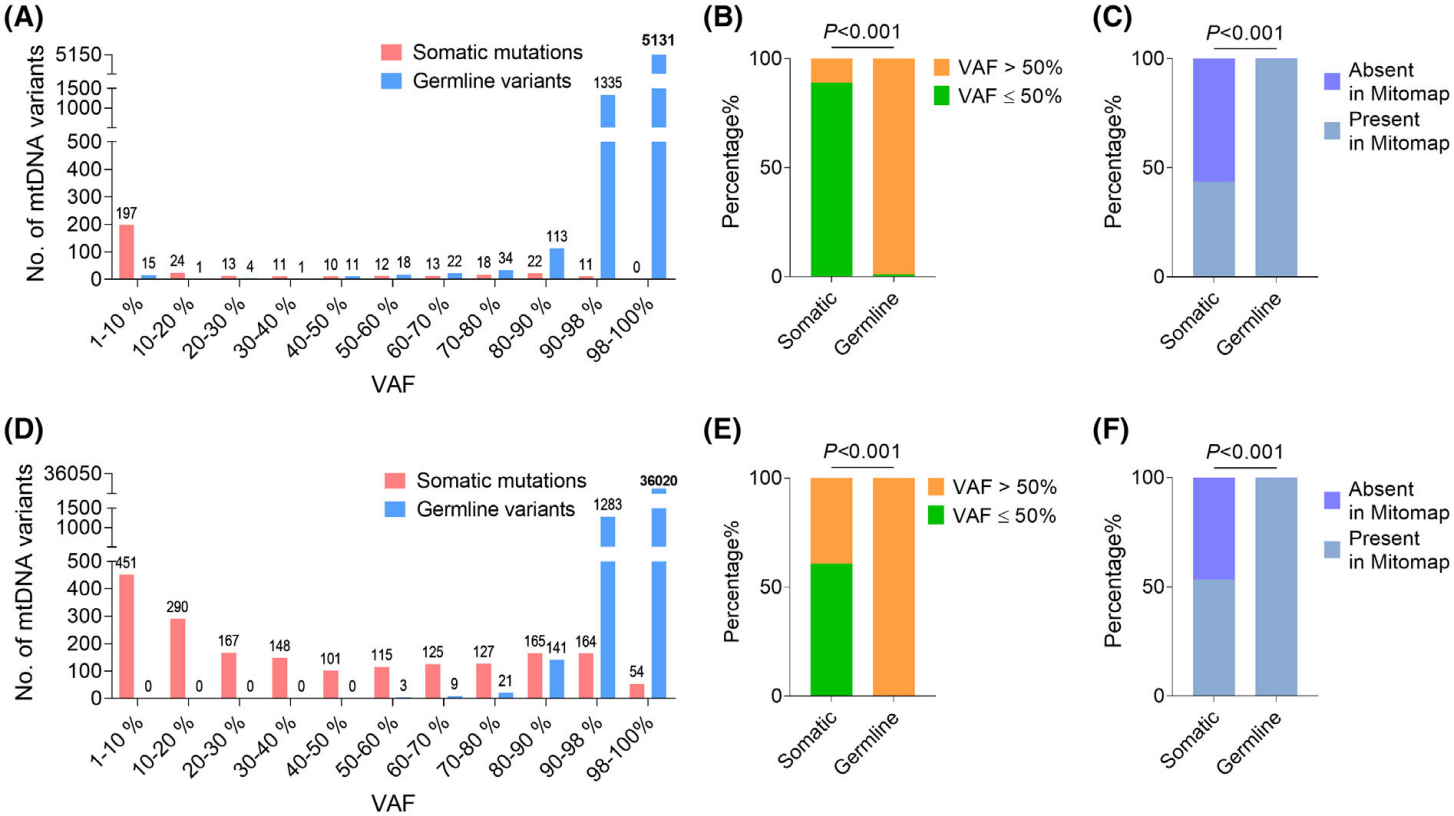
Comparison of the VAF and Mitomap status between somatic and germline mtDNA mutations. The VAF distribution of somatic (pink) and germline mtDNA variants (blue) in 199 private (A) and 1675 pan‐cancer (D) tumor samples. Distribution of somatic and germline variants based on 50% VAF cutoff in private (B) and pan‐cancer (E) samples. The occurrence of mtDNA variants in human population for private (C) and pan‐cancer (F) tumor samples. *P* values were calculated using the Chi‐square test. VAF, variant allele frequency.

We first analyzed the VAF distribution of mtDNA somatic and germline variants in private and pan‐cancer tumor samples. VAF of somatic mutations was inclined to be lower than 50%, whereas VAF of germline variants was mostly higher than 50% (Fig. 2A,B,D,E). In addition, almost 50% of mtDNA somatic mutations were absent in human Mitomap database, whereas less than 1% of mtDNA germline variants were absent in Mitomap (Fig. 2C,F). These results suggest that VAF of mtDNA variants and their occurrence in human population may be informative features for identifying somatic and germline mtDNA variants.

### 3.2 Optimization of the mitoSomatic classification algorithm

**Fig. 3:**
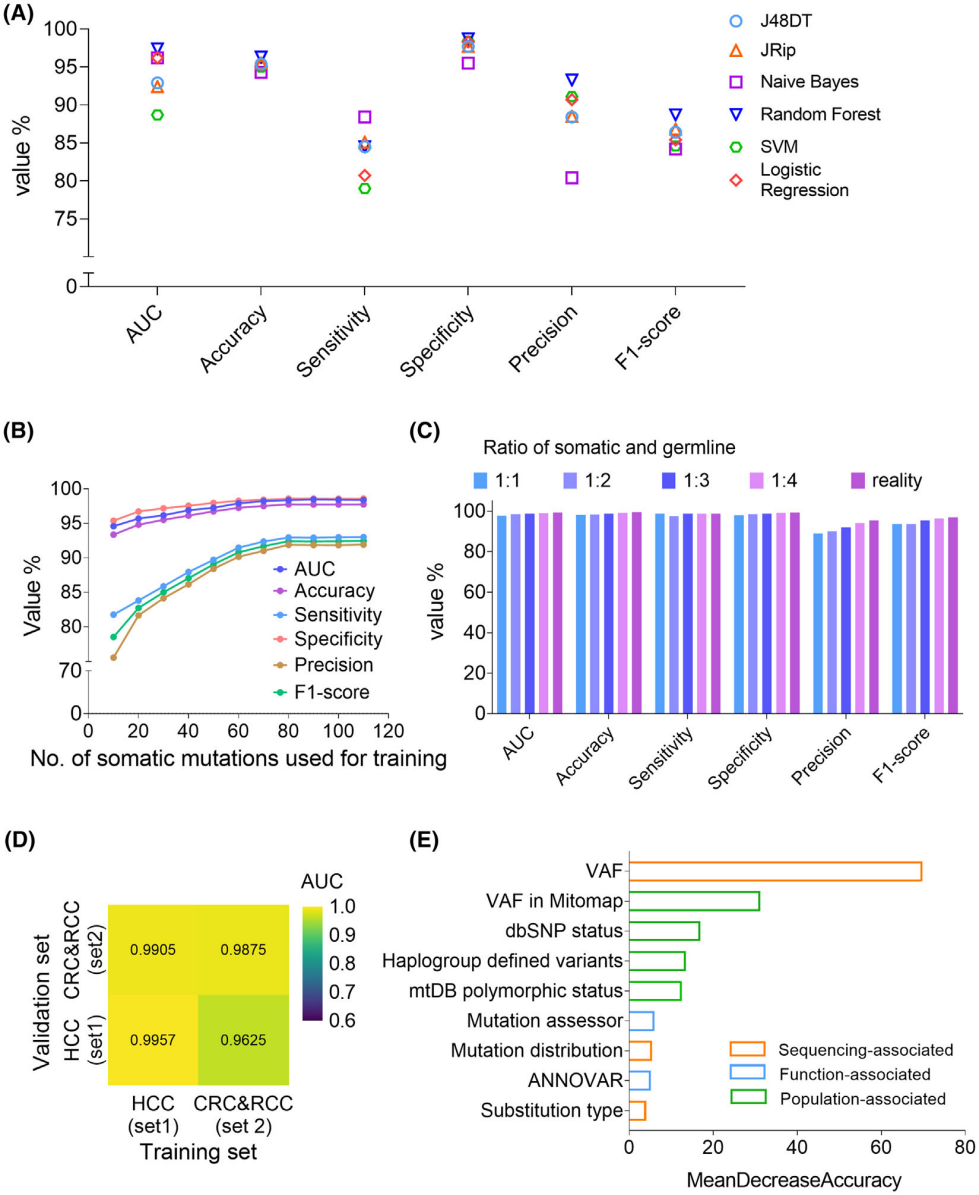
Optimization of the mitoSomatic classification algorithm. (A) Selection of optimal machine learning model using ground truth mtDNA variants in 157 HCC tumor samples. Six models as indicated were evaluated. (B) The effect of training set size on mitoSomatic performance. mitoSomatic was trained with a gradually increasing number of somatic mutations (*X*‐axis) and proportional germline variants in training set 1 (*n* = 10 independent repeats). (C) The effect of somatic and germline ratio in training set on mitoSomatic performance. Four training sets, composed of constant somatic (118 variants) but varying ratios of germline variants (1 : 1, 1 : 2, 1 : 3, 1 : 4) from training set 1, were evaluated using six evaluation metrics (*n* = 10 independent repeats). (D) The effect of cancer type within the training set on mitoSomatic performance. mitoSomatic was trained using one cancer type and tested on the same or another cancer type (*n* = 10 independent repeats). (E) Feature importance evaluation for mitoSomatic by the mean decrease in accuracy. AUC, area under the curve; dbSNP, the single nucleotide polymorphism database; mtDB, human mitochondrial genome database; SVM, support vector machine; VAF, variant allele frequency.

We first compared six supervised learning algorithms using six evaluation metrics. We found that random forest was optimal for classifying mtDNA somatic and germline variants (Fig. 3A). Analysis of training set size revealed that the performance of mitoSomatic initially improved with increasing training set size, but reached plateau stage when the number of somatic mutations exceeded eighty (Fig. 3B). Analysis of somatic and germline ratio in training set revealed that increasing germline variant proportion could moderately improve precision and F1‐score, but had little effect on other indicators. Accordingly, realistic somatic and germline variants ratio exhibited optimal overall performance, which we used at last (Fig. 3C). Analysis of cancer type in training set revealed that mitoSomatic showed no performance bias even if the training and test sets were inconsistent in cancer type, indicating that mitoSomatic performance is almost independent on the cancer type in the training set (Fig. 3D). Feature importance analysis showed that the mtDNA VAF and their frequency in human population were among the most important features for identification of mtDNA somatic mutations in mitoSomatic (Fig. 3E).

### 3.3 mitoSomatic performance for classification of mtDNA somatic mutations in tumor tissue

**Fig. 4:**
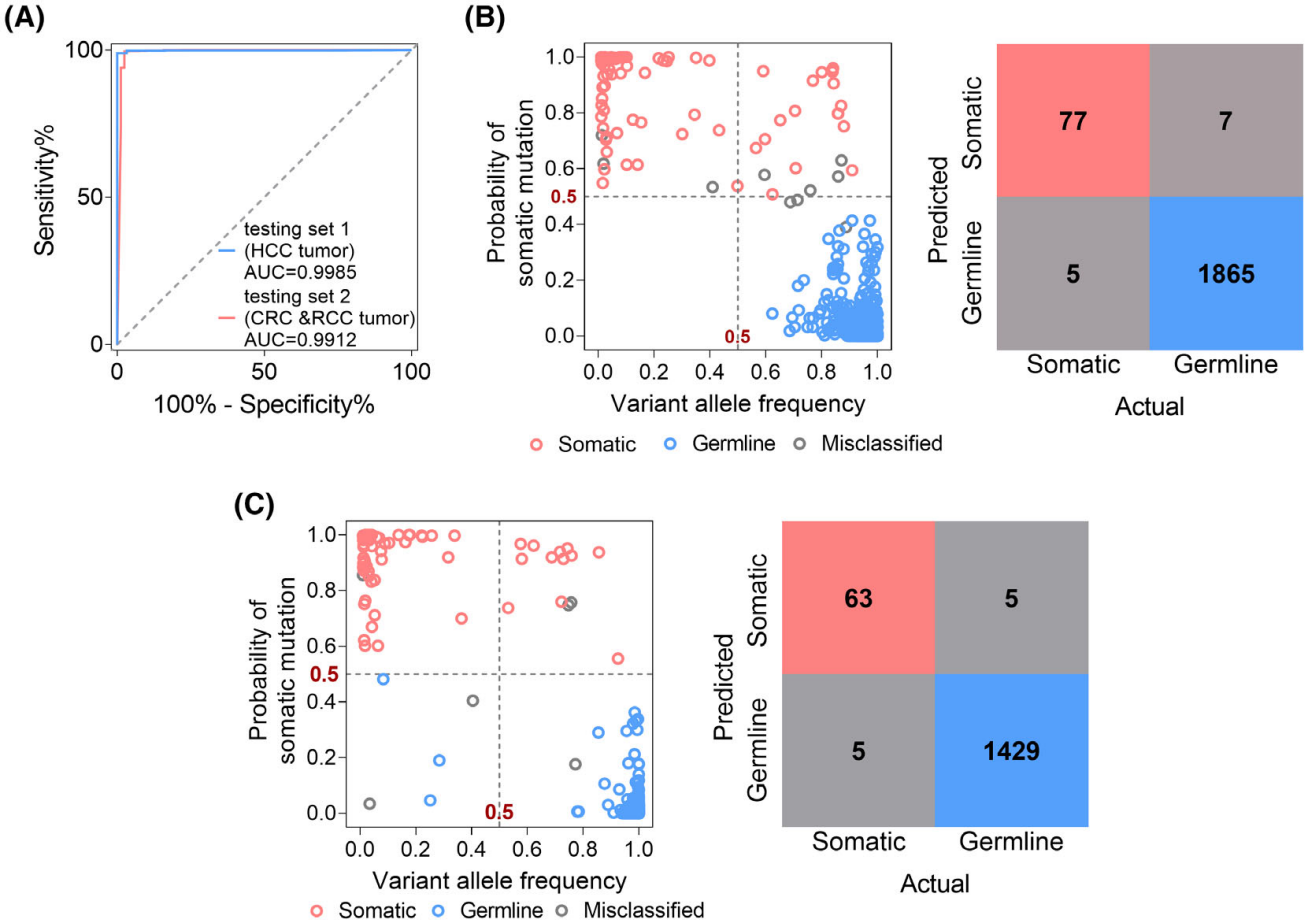
mitoSomatic performance for classification of mtDNA somatic and germline variants in 57 HCC tumor samples (testing set 1) and 24 CRC plus 18 RCC tumor samples (testing set 2). (A) ROC curves in testing sets 1 and 2. (B, C) mitoSomatic majority voting (left) and confusion matrix (right) analysis of somatic mutations in testing sets 1 and 2. Probability score of ≥ 0.5, somatic; otherwise, germline. Pink, blue, and gray circles denote somatic, germline, and misclassified variants, respectively. AUC, area under the curve.

We evaluated the performance of mitoSomatic using two independent testing sets, with testing set 1 containing 1954 mtDNA variants from 57 HCC samples, and testing set 2 containing 1502 mtDNA variants from 24 CRC and 18 RCC samples. In these two testing sets, mitoSomatic achieved AUC values of 0.9983 and 0.9912 for the classification of somatic mutations, respectively (Fig. 4A). Analysis of confusion matrixes revealed that mitoSomatic majority voting (probability of somatic mutation ≥ 0.5) accurately detected 93.9% somatic (77 out of 82) and 99.6% germline variants (1865 out of 1872) in testing set 1 (Fig. 4B). Meanwhile, it also accurately detected 92.6% somatic (63 out of 68), and 99.7% germline variants (1429 out of 1434) in testing set 2 (Fig. 4C). These results indicated that mitoSomatic accurately classified somatic and germline mtDNA variants in tumor tissue.

### 3.4 mitoSomatic performance on identification of mtDNA somatic mutations in tumor tissue without paired controls

**Fig. 5:**
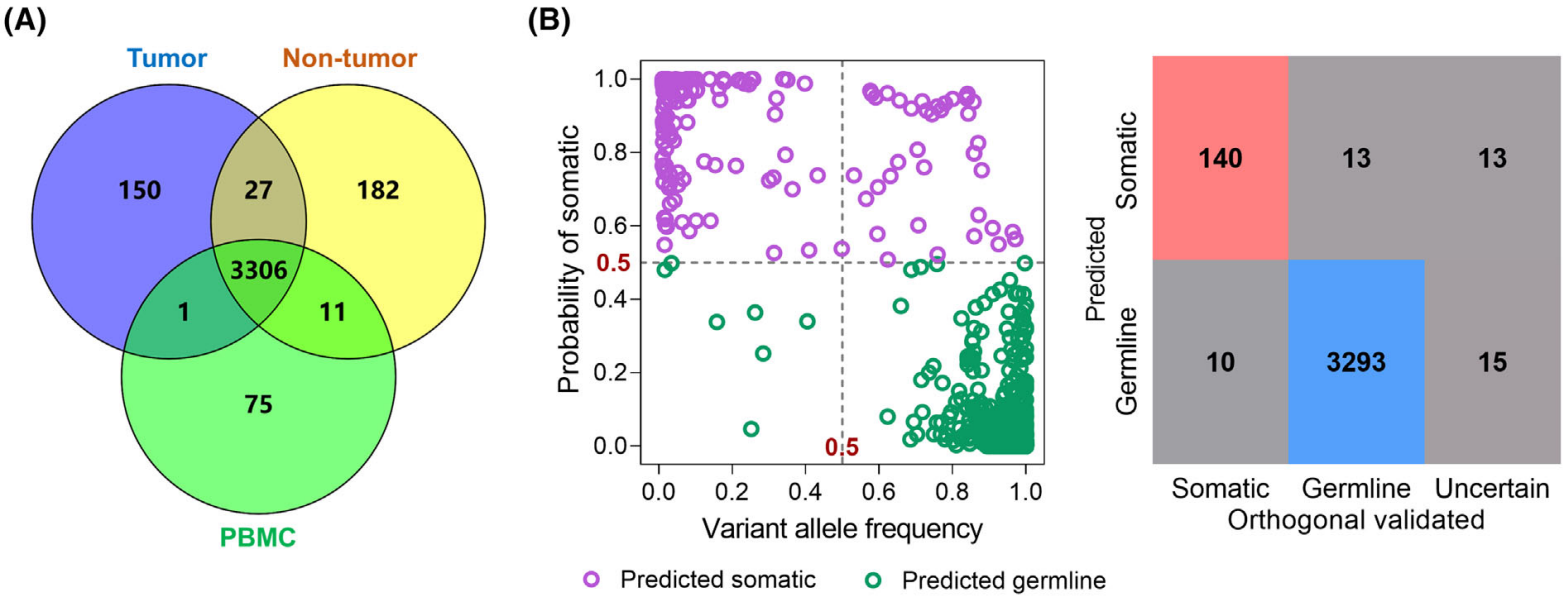
mitoSomatic performance on identification of somatic mtDNA variants in tumor tissue without paired controls. (A) Venn diagram of mtDNA variants detected in tumor, adjacent nontumor, and PBMC samples from 99 patients (57 HCC, 24CRC, and 18 RCC) (B) Majority voting (left) and confusion matrix (right) showing mitoSomatic prediction of all mtDNA variants in 99 tumor tissues. The somatic variants with a probability of ≥ 0.5 were identified as somatic, and germline variants with a probability < 0.5 were identified as germline, or misclassified otherwise. Red, blue, and gray boxes denote somatic, germline, and misclassified and uncertain variants, respectively. The accuracy of prediction was assessed according to the Venn diagram map. PBMC, peripheral blood mononuclear cell.

We next evaluated mitoSomatic performance on identification of mtDNA somatic mutations without paired controls based on 99 tumor tissues (57 from HCC, 24 from CRC, and 18 from RCC patients), which had both adjacent nontumor and PBMC samples as controls to provide ground truth somatic and germline mtDNA variants. In total, 3484 mtDNA variants were detected in these 99 tumor tissues, with 150 verified as somatic, 3306 as germline, and 28 as uncertain origin, respectively (Fig. 5A). mitoSomatic accurately identified 93.3% (140 out of 150) ground truth somatic mutations and 99.6% (3293 out of 3306) germline variants, respectively (Fig. 5B).

### 3.5 Evaluation of mitoSomatic for identification of somatic mutations in adjacent nontumor tissues or PBMCs

**Fig. 6:**
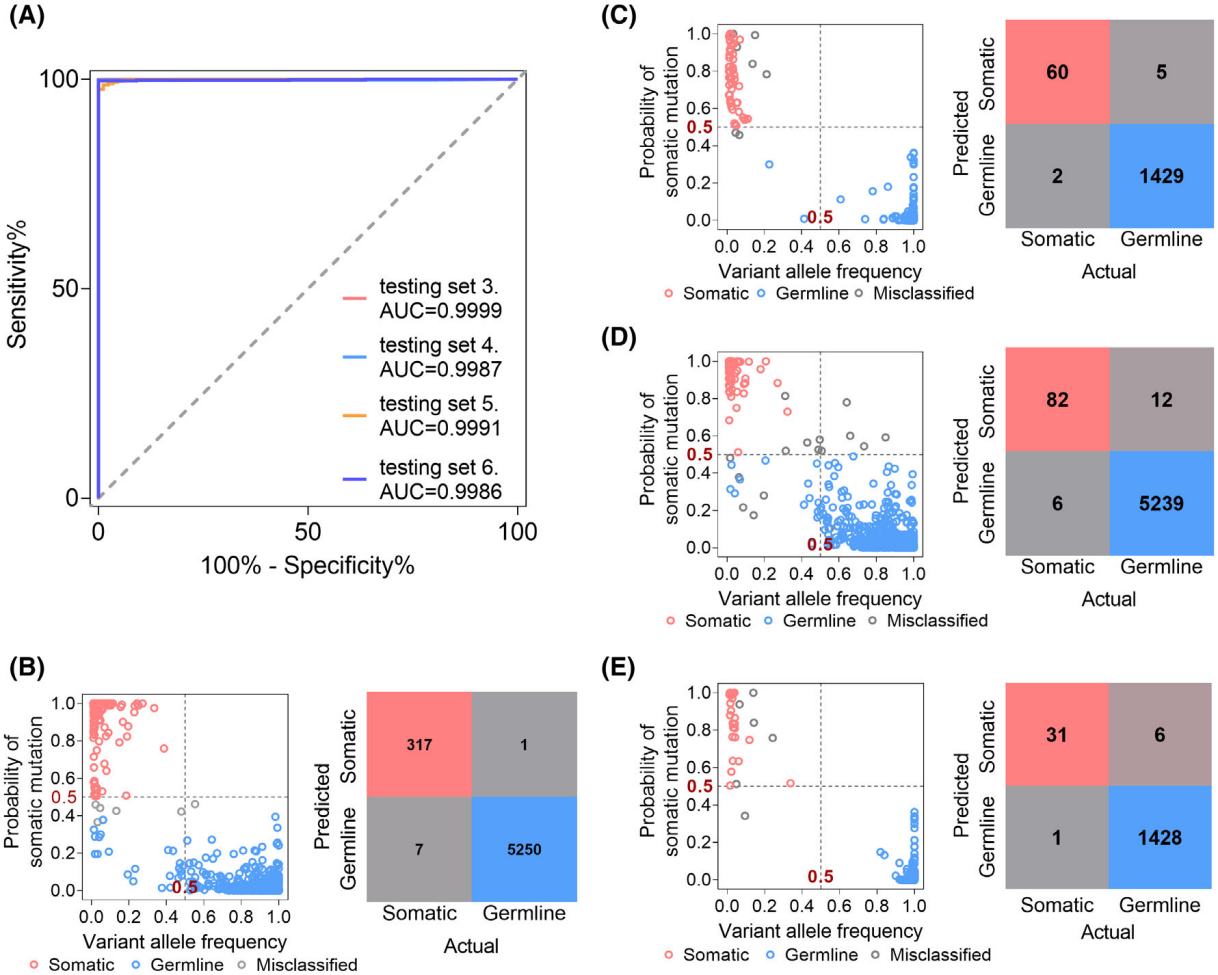
mitoSomatic performance on classifying mtDNA somatic and germline variants in adjacent nontumor or PBMC samples without paired control. Ground truth mtDNA variants in 157 HCC adjacent nontumor and PBMC samples were used as testing sets 3 and 4, and those in 24 CRC and 18 RCC adjacent nontumor and PBMC samples were used as testing sets 5 and 6, respectively. (A) ROC curves in testing sets 3–6. (B–E) Majority voting (left) and confusion matrix (right) showing mitoSomatic prediction of mtDNA variants in testing sets 3–6. The somatic mutations with a probability of ≥ 0.5 were identified as somatic, and germline mutations with a probability <0.5 were identified as germline, or misclassified otherwise. Red, blue, and gray circles and boxes denote somatic, germline, and misclassified variants, respectively. AUC, area under the curve.

The Venn diagram of the triple‐paired tumor, adjacent nontumor, and PBMC samples (Fig. S1) indicated the presence of mtDNA somatic mutations in nontumor tissues. Thus, based on testing sets 3–6, we further investigated whether mitoSomatic was applicable for identifying somatic mutations in nontumor sources. For all testing sets, mitoSomatic achieved AUC values over 0.99 (Fig. 6A) for the classification of somatic mutations. Confusion matrix analysis revealed that mitoSomatic correctly identified 97.8% (317 out of 324), 93.2% (82 out of 88), 96.8% (60 out of 62), and 96.9% (31 out of 32) somatic mutations, as well as 99.9% (5250 out of 5251), 99.8% (5239 out of 5251), 99.7% (1429 out of 1434), and 99.6% (1428 out of 1434) germline variants in testing sets 3–6, respectively (Fig. 6B–E). Overall, mitoSomatic yielded satisfying results in nontumor tissue sources, highlighting the flexibility of mitoSomatic's classification capability.

### 3.6 Evaluation of mitoSomatic for classifying mtDNA variants of uncertain germline or somatic origins

**Fig. 7:**
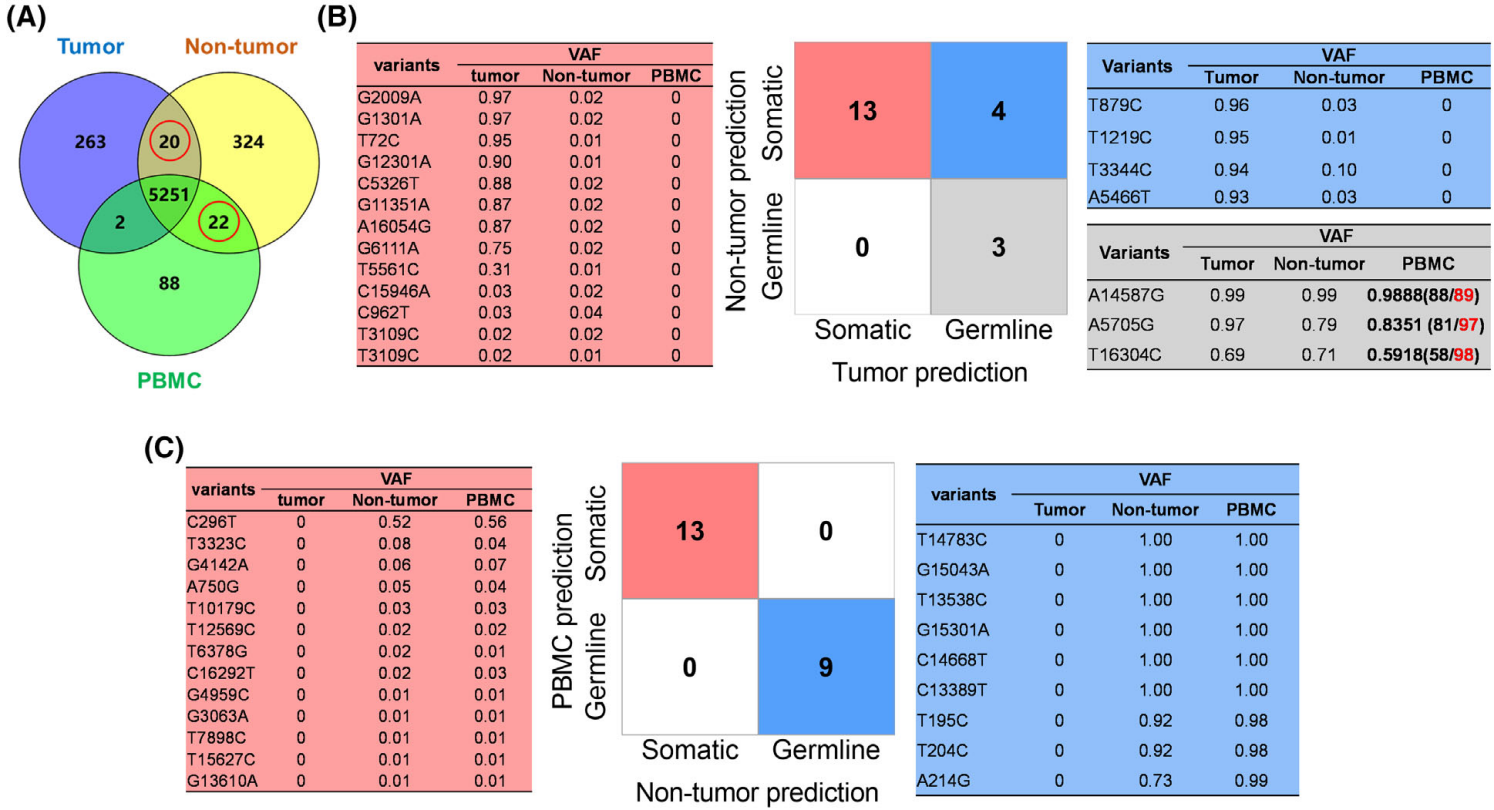
Evaluation of mitoSomatic for classifying mtDNA variants of uncertain germline or somatic origins in tumor, adjacent nontumor, and PBMC samples from 157 HCC patients. (A) Venn diagram of mtDNA variants in triple‐paired samples with uncertain variants red‐circled. (B) mitoSomatic prediction of 20 uncertain mtDNA variants shared by tumor and adjacent nontumor but absent in PBMC. (C) mitoSomatic prediction of 22 uncertain mtDNA variants shared by adjacent nontumor and PBMC but absent in tumor. The variants and corresponding VAF in triple‐paired samples are shown for each prediction class. PBMC, peripheral blood mononuclear cell; VAF, variant allele frequency.

Conventionally, either adjacent non‐tumor or PBMC sample was used as single control for calling somatic mutations in tumor, with germline variants defined as variants shared in both tumor and paired control. Nevertheless, the Venn diagram of the triple‐paired tumor, adjacent nontumor, and PBMC samples revealed a small number of mtDNA variants with uncertain germline or somatic origin, as such variants were only observed in two tissue types but absent in the third paired sample (Fig. 7A; Table S3). Thus, mitoSomatic was applied to these mtDNA variants of uncertain origin to predict their possible source.

Of the 20 uncertain variants shared by tumor and adjacent nontumor but absent in PBMC samples, three were predicted as germline, and thirteen were predicted as somatic in both tumor and adjacent nontumor. Still, the remaining four variants were predicted inconsistently to be somatic in adjacent nontumor and germline in tumor (Fig. 7B). Combined examination of tumor, adjacent nontumor, and PBMC sequencing data revealed that all three variants predicted as germline in tumor and adjacent nontumor could actually be observed in PBMC with comparable VAF, but were filtered out due to lower sequencing depth in PBMC (< 100×), thus supporting their germline prediction (Fig. 7B, right panel). Consistent with somatic origins, all the 13 variants predicted as somatic in both tumor and adjacent nontumor were undetectable in PBMC samples, but nine of them exhibited high VAF in tumor but low VAF (~ 1%) in adjacent nontumor, suggesting either contamination of tumor cells into adjacent nontumor or dramatic VAF shift between tumor and adjacent nontumor tissues (Fig. 7B, left panel). The remaining four variants received inconsistent prediction in tumor and adjacent nontumor samples. Still, they were undetected in PBMC samples and were not cataloged in dbSNP or Mitomap databases, indicating that they were more likely to be somatic.

Variants shared between two developmentally distinct tissues, such as PBMC and adjacent nontumor, were more likely germline in origin. However, mitoSomatic analysis of the 22 uncertain variants present in adjacent nontumor and PBMC yielded consistent prediction, with nine as germline and thirteen as somatic (Fig. 7C). Detailed examination of the sequencing data revealed that none of these 22 variants were detectable in tumor, but nearly all nine predicted germline variants were high‐frequency variants (VAF > 90%) in both PBMC and adjacent nontumor (Fig. 7C, right panel) while almost all 13 predicted somatic mutations were low‐frequency variants (VAF ~ 1%) in both PBMC and adjacent nontumor (Fig. 7C, left panel). Although no conclusive evidence is available, these variants present in PBMC and adjacent nontumor but absent in tumor could reflect downward VAF drifting/shifting in tumor tissue due to continuous proliferation.

### 3.7 mitoSomatic evaluation of mtDNA somatic mutations in published pan‐cancer dataset

**Fig. 8:**
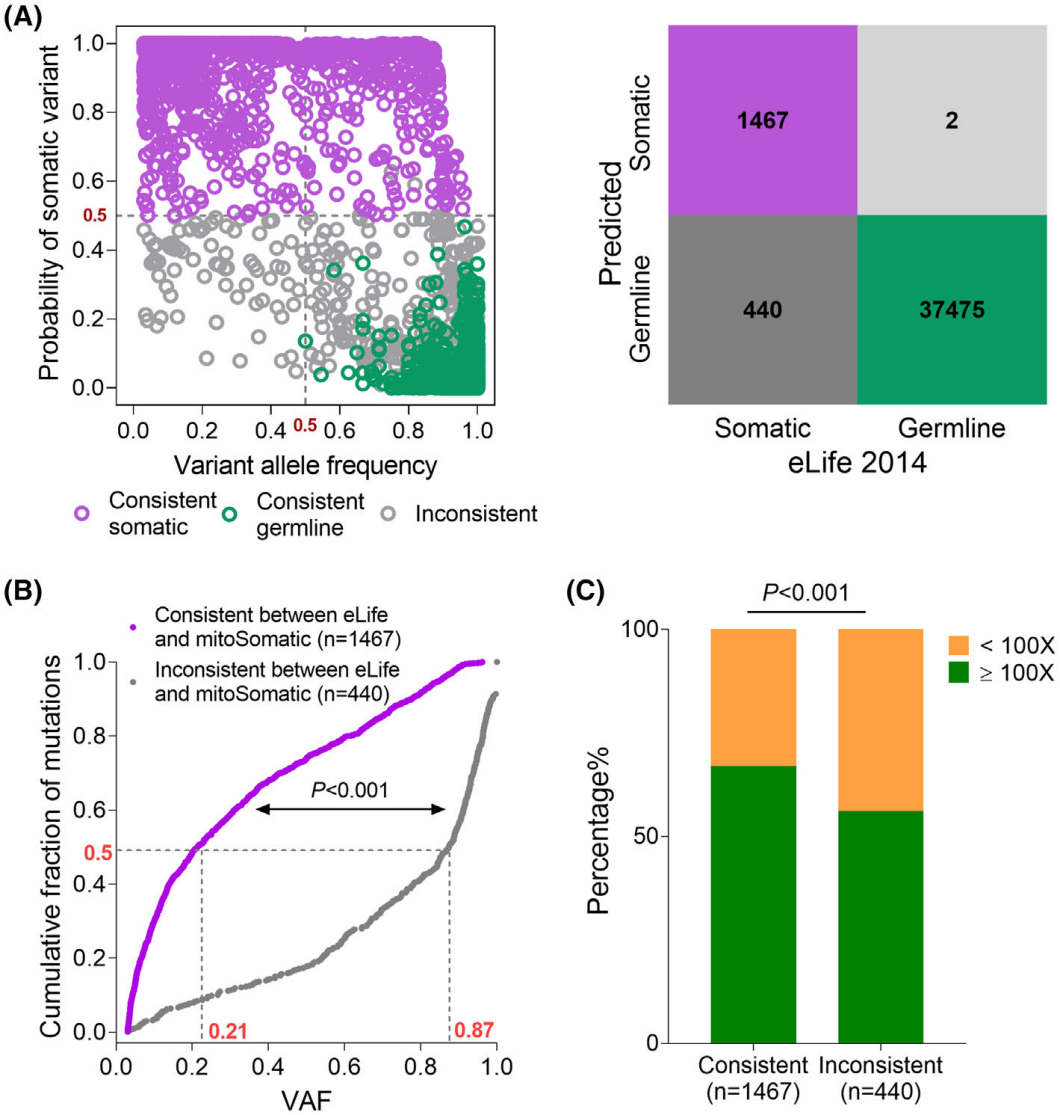
mitoSomatic evaluation of mtDNA somatic mutations in a published pan‐cancer dataset. (A) Majority voting (left) and confusion matrix (right) comparison of mtDNA variants in 1675 pan‐cancer samples from *elife* dataset [5]. mitoSomatic predictions were compared with the *eLife* labeling: consistent somatic (purple), consistent germline (green), and inconsistent (gray). (B) Cumulative distributions of VAF for *eLife*‐labeled somatic mutations with consistent (1467) or inconsistent (440) mitoSomatic prediction. *P*‐value, two‐sample Kolmogorov–Smirnov test. (C) Distinct distribution of sequencing depth for *eLife*‐labeled somatic mutations with consistent or inconsistent mitoSomatic prediction based on 100× site depth cut‐off in adjacent nontumor. VAF, variant allele frequency. *P*‐value, Chi‐square test.

Finally, we applied mitoSomatic to analyze mtDNA somatic mutations in a pan‐cancer dataset consisting of 1675 tumor‐control pairs from 31 tumor types published in *eLife* [5], in which a total of 1907 somatic and 38 706 germline mtDNA variants were assigned based on matched tumor‐control comparison. Notably, mitoSomatic achieved highly consistent prediction for the originally assigned germline variants (Fig. 8A). However, of the 1907 originally assigned somatic mutations, mitoSomatic analysis revealed significant inconsistency by predicting 440 of them as germline (Fig. 8A).

In addition, further analysis of the 440 suspicious ‘somatic’ mtDNA variants revealed a dramatically deviated VAF distribution, with more than 50% of variants having a VAF higher than 87% (*P* < 0.001, Fig. 8B). As tumor tissues are mostly mixtures of malignant cells and normal stromal cells, this extreme enrichment of high VAF variants probably indicated that a high proportion of them are more likely to be germline. Furthermore, nearly half of the 440 suspicious ‘somatic’ mtDNA variants (43.9%, 193 out of 440) had a site sequencing depth below 100× in adjacent non‐tumor tissues (Fig. 8C), supporting that the corresponding variants in adjacent nontumor tissue may be filtered out due to low sequencing depth, thus leading to false‐positive somatic mutations in paired tumor tissues. These results demonstrated that, even with a matched control available, mtDNA somatic mutations could not always be correctly identified in tumor tissue, emphasizing the usefulness of mitoSomatic in real‐world NGS data.

## 4 Discussion

In the present study, a novel machine learning tool, mitoSomatic, was for the first time developed to accurately identify somatic and germline variants in mtDNA from various tumor tissues, with a unique advantage of being applicable even without paired controls. The remarkable performance of mitoSomatic was also extended to adjacent nontumor tissues and PBMCs, indicating its generalized application. More importantly, this tool will significantly improve the accuracy of identifying mtDNA variants' somatic/germline origin, which can provide a better understanding of mtDNA mutational profiles.

Conventionally, tumor‐specific (somatic) mutations were identified by data comparison between tumor and paired control tissues, commonly adjacent nontumor tissue or PBMC, to filter out the shared germline variants in NGS sequencing data [5, 6, 34]. In such circumstances, the accuracy of identifying somatic/germline origin greatly depends on the variant calling in both paired tissues. Once false‐negative calling occurs in paired controls, germline variants will be misclassified as somatic in tumor tissues. In diploid nuclear genomes, it is relatively easy to identify germline variants given their characteristics of either heterozygous (50% VAF) or homozygous (100% VAF). However, high copy number of mtDNA in a single cell leads to the heteroplasmy of mtDNA variants [35], which in conjunction with NGS‐associated sequencing bias, could complicate the identification of germline variants with low frequency, especially in paired control tissues, thus leading to false positive somatic mutation calling in tumor tissues. Consistent with this rationale, the reanalysis of previously published pan‐cancer data [5] by mitoSomatic identified a substantial number of suspicious ‘somatic’ variants, with supporting evidence of possible misclassification that a bias distribution toward high VAF (> 90%) existed in tumor tissues. If we consider that tumor tissues are mostly mixtures of malignant cells and surrounding stromal cells, mtDNA somatic mutations with extremely high VAF (> 90%) should be rare [6]. Thus, even in conditions where matched tumor‐normal tissues are available, mitoSomatic exhibits significant improvement in facilitating accurate calling of mtDNA somatic mutations. In addition, due to the pan‐cancer similarity and lack of tumor‐stage specificity for mtDNA mutation signatures, mitoSomatic is less feasible to identify the type and stage of tumor cells.

In clinical practices, using paired samples will greatly increase the economic and time costs, and limit the widespread application. To the best of our knowledge, there was no available approach to identify somatic/germline mtDNA variants in tumor tissues without paired controls. Random forest‐based machine learning is well known for integrating multidimensional features and processing large amounts of data [36]. Kalatskaya et al. [19] recently reported a random forest‐based approach for predicting somatic mutations in the nuclear genome without paired controls. However, considering the specific characteristics of mtDNA, this approach is unsuitable for identifying mtDNA somatic mutations. Integrating a set of nonredundant mtDNA variant features (VAF, occurrence in population and functional impact score, etc.) into random forest learning, we successfully developed a useful tool mitoSomatic to identify mtDNA somatic mutations accurately and cost‐effectively without paired controls.

Since mtDNA somatic and germline variants may function with great difference in tumorigenesis, it is important to accurately identify the somatic/germline origin of mtDNA variants. It is worth noting that our data analysis with the triple‐paired tumor, adjacent nontumor, and PBMC samples revealed a small group of mtDNA variants with uncertain somatic/germline origin, as these variants were only observed in two tissue types but absent in the third paired sample. Similarly, a recent pan‐tissue mtDNA mutation profiling study has also reported the extensive presence of mtDNA variants with uncertain origin, which are present in only two tissue types but absent from other tissues [14]. Based on the conventional comparison between tumor and control pairs, these uncertain variants will thus be classified as either somatic or germline variants depending on adjacent nontumor or PMBC as control, further showing the limitation of conventional paired samples in identifying mtDNA somatic mutations. Fortunately, our data showed that mitoSomatic could significantly improve the prediction of possible origin in mtDNA variants. Four potential sources were revealed by mitoSomatic analysis of mtDNA variants with uncertain origin in triple‐paired tumor, adjacent nontumor, and PBMC: (i) true germline variants with failed detection in PBMC due to low site sequencing depth (false negative in PBMC); (ii) impurity or contamination of tumor cells into adjacent nontumor tissues (false positive in adjacent nontumor); (iii) shared somatic mutations with heteroplasmy shifting (true somatic) between tumor and adjacent nontumor tissues; (iv) loss of heteroplasmic germline variants in tumor tissues due to drifting and continuous proliferation (downward shifting). Our results suggest the complexity of identifying the origin of mtDNA variants. Our data also indicated the possibility of SNP (germline variants) shift involved in tumorigenesis, which needs to be further verified in future studies.

## 5 Conclusions

mtDNA somatic mutations play essential roles in cancer. Although NGS sequencing of paired tumor and control samples is commonly used to identify mtDNA somatic mutations, this strategy is flawed due to mtDNA heterogeneity and is inapplicable for scenarios without matched normal tissue. Taken together, our work demonstrates that mitoSomatic is a valuable tool for accurate identification of mtDNA somatic mutations in mtDNA NGS data, applicable for both tumor and nontumor tissues without paired controls.

## Conflict of interest

The authors declare no conflict of interest.

## Author contributions

JX conceived and designed the study. WG and YL carried out the sample collection, performed the data analysis, and drafted the manuscript. LS, SG, and FX helped with algorithm design and implementation. XJ and KZ performed the laboratory experiments. XG participated in the study's design and revised the manuscript draft. JX and XG conceived, designed, and coordinated the study, carried out the analysis, and drafted and revised the manuscript. All authors read and approved the final manuscript.

## Supporting information


**Fig. S1.** Venn diagrams of mtDNA variants detected in the tumor, nontumor, and PBMC samples from 157 HCC (A), 24 CRC (B), and 18 RCC (C) patients.
**Table S1.** Summary of mtDNA capture‐based NGS data.
**Table S2.** Catalog of mtDNA variants in 157 HCC, 24 CRC, and 18 RCC patients.
**Table S3.** mtDNA uncertain variants in 157 HCC patients.
**Table S4.** Details for training, validation, and testing sets.
**Table S5.** List of features used in mitoSomatic.

## Data Availability

The public mtDNA NGS data in this study are available in *eLife* 2014 [5]. The mtDNA variants data generated in our lab are available in the National Genomics Data Center (NGDC) Genome Variation Map (GVM) database (https://ngdc.cncb.ac.cn/gvm/) under accession number GVM000346. The mitoSomatic package is available on https://github.com/3150129019/mitoSomatic. The package is written in the r (version 4.0.5) programming language and is actively maintained. Further information is available from the corresponding authors upon request.
