# Supplementary material for: mitoSomatic: a tool for accurate identification of mitochondrial DNA somatic mutations without paired controls
Source: Mol Oncol. 2022 Dec 15;17(5):857–71. doi: 10.1002/1878-0261.13335 (PMC10158781; doi:10.1002/1878-0261.13335)
Supplement: Supplementary file 1 — Fig. S1. Venn diagrams of mtDNA variants detected in the tumor, nontumor, and PBMC samples from 157 HCC (A), 24 CRC (B), and 18 RCC (C) patients. Table S1. Summary of mtDNA capture‐based NGS data. Table S2. Catalog of mtDNA variants in 157 HCC, 24 CRC, and 18 RCC patients. Table S3. mtDNA uncertain variants in 157 HCC patients. Table S4. Details for training, validation, and testing sets. Table S5. List of features used in mitoSomatic. [file MOL2-17-857-s001.docx]

**mitoSomatic: a tool for accurate identification of mitochondrial DNA somatic mutations without paired controls**

Wenjie Guo^1#,^ Yang Liu^1#^, Liping Su^1^, Shanshan Guo^1^, Fanfan Xie^1^, Xiaoying Ji^1^, Zhoukai Xiang^1^, Xu Guo^1^, Xiwen Gu^2^*, Jinliang Xing^1^*

**Contents**

**Supplementary material includes 1 figure and 5 tables:**

**Fig. S1.** Venn diagrams of mtDNA variants detected in the tumor, non-tumor, and PBMC samples from 157 HCC (A), 24 CRC (B), and 18 RCC (C) patients.

**Table S1.** Summary of mtDNA capture-based NGS data.

**Table S4.** Details for training, validation, and testing sets.

**Table S5.** List of features used in mitoSomatic.

**Note**: the **Table S2** and **Table S3** are presented separately.

**Supplementary figure**

**
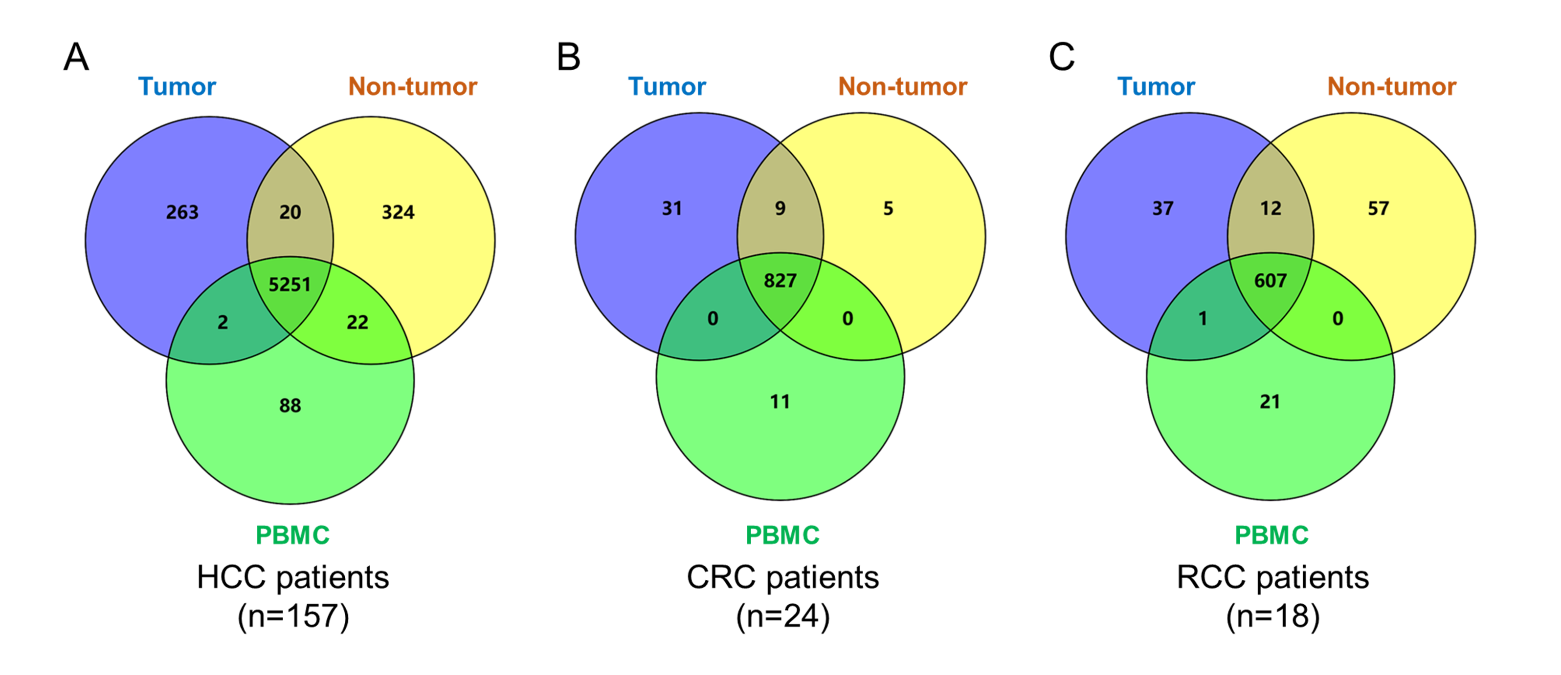
**

**Fig. S1.** Venn diagrams of mtDNA variants detected in the tumor, non-tumor, and PBMC samples from 157 HCC (A), 24 CRC (B), and 18 RCC (C) patients.

**Supplementary tables**

| **Table S1.** Summary of mtDNA capture-based NGS data. | | | | | |
| --- | --- | --- | --- | --- | --- |
| **Patients (n)** | **Sample type** | **Average depth (×)** | **Average coverage** | **Average Q30** | **Average chrM mapping rate** |
| HCC  (157) | Tumor | 9011±4575 | 100% | 93.24% | 77.68% |
|  | Non-tumor | 8656±4817 | 100% | 92.34% | 80.32% |
|  | PBMC | 4778±3689 | 100% | 93.89% | 79.56% |
| CRC  (24) | Tumor | 4675±1799 | 100% | 93.73% | 67.13% |
|  | Non-tumor | 4886±1791 | 100% | 94.12% | 64.55% |
|  | PBMC | 2015±792 | 100% | 93.67% | 45.29% |
| RCC  (18) | Tumor | 4925±2537 | 100% | 95.25% | 65.01% |
|  | Non-tumor | 6113±2487 | 100% | 96.09% | 66.67% |
|  | PBMC | 1477±663 | 100% | 94.73% | 41.94% |
| **Note:** HCC, hepatocellular carcinoma; RCC, renal cell carcinoma; CRC, colorectal cancer; PBMC, peripheral blood mononuclear cell. | | | | | |

| **Table S4**. Details for training, validation, and testing sets. | | | | |
| --- | --- | --- | --- | --- |
| **Data sets** | **Tumor type (n)** | **Total variants** | **No. of somatic** | **No. of germline** |
| Training set 1* | HCC tumor (70) | 2501 | 118 | 2383 |
| Validation set 1* | HCC tumor (30) | 1059 | 63 | 996 |
| Training set 2^△^ | CRC (18) & RCC (13) tumor | 1203 | 51 | 1074 |
| Validation set 2^△^ | CRC (6) & RCC (5) tumor | 299 | 17 | 358 |
| Testing set 1* | HCC tumor (57) | 1854 | 82 | 1872 |
| Testing set 2 | CRC (24) & RCC (18) tumor | 858 | 68 | 1434 |
| Testing set 3 | HCC non-tumor (157) | 5575 | 324 | 5251 |
| Testing set 4 | HCC PBMC (157) | 5339 | 88 | 5251 |
| Testing set 5 | CRC (24) & RCC (18) non-tumor | 832 | 62 | 1434 |
| Testing set 6 | CRC (24) & RCC (18) PBMC | 838 | 32 | 1434 |
| **Note:** *The total number of HCC tumor=157; ^△^The total number of CRC tumor=24, the total number of RCC tumor=18. | | | | |

| **Table S5.** List of features used in mitoSomatic. | | | | |
| --- | --- | --- | --- | --- |
| **Features** | **Classes** | **Description** | **Number**  **of values** | **Type**  **of value** |
| dbSNP status | Population-associated | A free public archive for germline variants. | 2 | Boolean |
| VAF in Mitomap | Population-associated | The frequency of a specific variant in the Mitomap database, ranging from 0 to 1. | Numerical | Double |
| Haplogroup-defining variants | Population-associated | Haplogroup-defining variants are stable mtDNA polymorphisms that identify individuals into mitochondrial haplogroups. | 2 | Boolean |
| mtDB polymorphic status | Population-associated | mtDB, Human Mitochondrial Genome Database, a resource for population genetics and medical sciences. | 2 | Boolean |
| VAF | Sequencing-associated | The fraction of mutant reads in the total reads for a specific variant site, ranging from 0 to 1. | Numerical | Double |
| Mutation distribution | Sequencing-associated | mtDNA was divided into four different regions: D-loop, protein-coding, non-protein coding, and another region. | 4 | Categorical |
| Substitution type | Sequencing-associated | The allele substitution from the reference to the variant allele. | 12 | Categorical |
| ANNOVAR | Function-associated | A software to functionally annotate pathogenic effect of variants. | 5 | Categorical |
| Mutation assessor | Function-associated | A predictor of the functional impact of amino acid substitutions in proteins. | 5 | Categorical |
